# High Serum Lipase Level is Associated With Increased Analgesic Use in Hospitalized Patients With Acute Pancreatitis

**DOI:** 10.1155/prm/6462957

**Published:** 2026-03-08

**Authors:** Liangqing Gao, Ruifang Zhang, Yizhi Xiao, Chengmin Ma, Xiaofeng Li

**Affiliations:** ^1^ Department of Gastroenterology, The Fifth Affiliated Hospital, Sun Yat-sen University, Zhuhai, China, sysu.edu.cn

## Abstract

**Background:**

Although pain management plays an important role in the treatment of acute pancreatitis (AP), current guidelines lack clear and consistent recommendations for the management of abdominal pain. The aim of this study was to investigate factors associated with analgesic use in Chinese hospitalized patients with AP.

**Methods:**

This was a single‐center retrospective study. Patients discharged with a diagnosis of AP were consecutively included. Patients were divided into the analgesic group and the nonanalgesic group based on whether they received analgesic therapy. Clinical parameters, including baseline pain scores and serum lipase level, were compared using univariate analysis and multivariate logistic regression.

**Results:**

A total of 151 patients were included, with 69 (45.7%) receiving analgesic treatment and 82 (54.3%) receiving no analgesics. Patients with moderate‐to‐severe pain (score 4–7) at admission were 6.86 times more likely to receive analgesics than pain‐free patients (95% confidence interval [CI]:1.12–41.99, *p* = 0.037). Moderately severe and severe AP (vs. mild) were associated with 2.94‐fold (odds ratio [OR] = 3.94, 95% CI: 1.19–12.98, *p* = 0.024) and 4.05‐fold (OR = 5.05, 95% CI: 1.22–20.8, *p* = 0.025) increased risk of analgesic use, respectively. Although 108.1 U/L ≤ lipase level < 293.4 U/L and 293.4 U/L ≤ lipase level < 809.6 U/L (vs.< 108.1 U/L) did not significantly increase analgesic use risk, patients with lipase level ≥ 809.6 U/L had a 2.8‐fold increased risk (OR = 3.8, 95% CI: 1.27–11.37, *p* = 0.017).

**Conclusions:**

Elevated serum lipase (≥ 809.6 U/L), higher baseline pain scores, and AP severity are key factors associated with analgesic use in Chinese AP patients. These findings highlight serum lipase as a potential biomarker for individualized pain management strategies in AP.

## 1. Background

Abdominal pain is one of the most prominent symptoms of acute pancreatitis (AP) [1]. Severe pain at admission is associated with an increased risk of severe AP and systemic or local complications [2, 3]. In patients with hemoconcentration at presentation, the duration of abdominal pain prior to admission may influence the severity of AP [4]. Early and effective pain relief can improve patient satisfaction, alleviate anxiety, and reduce the risk of complications such as respiratory compromise and thrombosis [5–7]. Although pain management plays an important role in the treatment of AP, current guidelines lack clear and consistent recommendations for the management of abdominal pain [8, 9]. In clinical practice, opioids and nonsteroidal anti‐inflammatory drugs (NSAIDs) are the most commonly used analgesics for AP [10, 11]. Studies have shown that NSAIDs and opioids are equally effective for pain relief in AP under specific circumstances [7, 12]. There are significant regional differences in the use of opioid analgesics for AP. In North America, over 90% of AP patients receive opioid analgesics, while in some regions, opioid use is less than 30% [13]. This reflects substantial regional variability in analgesic strategies for AP. Investigating factors related to the use of analgesics in patients with AP in specific regions is particularly meaningful and could help prevent underuse or overuse of analgesics, especially opioids, which will also provide clues for developing individualized pain management strategies. Previous studies have conducted similar investigations, but the study populations were U.S. patients [14]. Findings from U.S.‐based studies may not be directly applicable to non‐U.S. patient populations, given that the annual intensity of opioid use in the U.S. is over 80 times that of China [15]. The purpose of this study is to analyze factors associated with analgesic use in Chinese AP patients based on the disease and patient characteristics.

## 2. Methods

### 2.1. Study Design

This was a single‐center retrospective study and has been approved by the Ethics Review Committee of our hospital. Due to the retrospective and anonymous nature of the data, the informed consents were waived.

### 2.2. Inclusion and Exclusion Criteria

Patients discharged from our department with a diagnosis of AP from June 2022 to August 2023 were consecutively screened. and the patients aged 18 years or older were retrospectively included. Exclusion criteria were (1) pregnancy, (2) a history of chronic pancreatitis, (3) in‐hospital mortality, and (4) the clinical data required for collection is incomplete. AP was defined according to the revised Atlanta classification [16].

### 2.3. Sample Size Calculation

The sample size was calculated using the events per variable (EPV) metric [17]. EPV is widely used in binary logistic regression analysis, with the rule suggesting that each independent variable (predictor) should have at least 10 events (e.g., occurrences of the outcome) to ensure robustness and reliability of statistical results. Based on prior studies, 57.8% of patients with acute abdominal pain visiting the emergency department received analgesic treatment [6], and we estimated that 50% of patients in this study would receive analgesics. Given our intention to include seven predictor variables and set the EPV to 10, the required sample size was calculated as 140 cases. The complete calculation formula is as follows: sample size = number of variables × EPV/(1 − outcome incidence rate) = 7 × 10/(1 − 50%) = 140.

### 2.4. Data Collection

Data collected included all types and frequencies of analgesics used for pancreatic pain during hospitalization, admission time window (00:00–08:00, 08:00–16:00, 16:00–24:00), days since symptom onset, heart rate, blood pressure, body mass index (BMI), white blood cell (WBC) count, hemoglobin, alanine aminotransferase (ALT), aspartate aminotransferase (AST), total bilirubin (TBIL), C‐reactive Protein (CRP), triglycerides (TG), serum lipase, serum amylase, maximum pain score (0−10‐point numerical rating scale with “no pain” being 0 point and “worst pain imaginable” as 10 points) at baseline, first episode of AP or not, etiology and severity of AP, length of hospital stay, and education level. The disease severity collected here was obtained from discharge diagnoses and was not assessed either on admission or before analgesic administration. The analgesics administered in our hospital for pain caused by AP included opioids, NSAIDs, and others. Specifically, levo‐tetrahydropalmatine, bucinnazine, and antispasmodics were classified as “other” analgesics. Patients were divided into the analgesic group and the nonanalgesic group based on whether they received analgesic therapy.

### 2.5. Statistical Analysis

All statistical analyses were performed using IBM SPSS Statistics 29 and Microsoft Office Excel 2021. Normally distributed continuous data were expressed as mean ± standard deviation (x ± s), with group comparisons using independent‐sample *t*‐tests. Nonnormally distributed continuous data were expressed as median and interquartile range (IQR), with group comparisons using Wilcoxon rank‐sum tests. Categorical data were described using frequencies, percentages, or ratios, with group comparisons using chi‐square tests or Fisher’s exact test. Correlations between independent and dependent variables were analyzed using appropriate regression models: binary logistic regression was applied for dichotomous dependent variables, whereas multiple linear regression was used for continuous dependent variables. A *p* value < 0.05 was considered statistically significant.

## 3. Results

**FIGURE 1:**
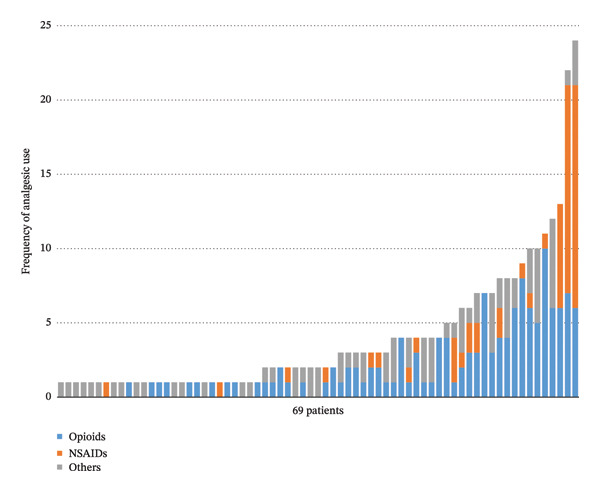
Distribution of usage of different analgesics in the analgesic group of 69 patients.

A total of 151 patients were included, with 69 (45.7%) receiving analgesic treatment and 82 (54.3%) receiving no analgesics (Table 1). Details of analgesics used in the 69 patients of the analgesic group are shown in Figure 1. There were no significant differences between the two groups in age, sex, BMI, admission time window, proportion of first episodes, blood pressure (including systolic and diastolic pressure), TBIL, transaminases (including ALT and AST), serum amylase, CRP, or education level. Significant differences were observed in baseline pain scores (*p* = 0.029), with a larger proportion of patients scoring 4–7 in the analgesic group (23.2% vs. 9.8% in the nonanalgesic group). Significant differences were also noted in etiology distribution (*p* = 0.041): hypertriglyceridemia‐associated pancreatitis was most common in the analgesic group (37.7%), while “other causes” predominated in the nonanalgesic group (52.4%). Although blood pressure at admission was similar between the two groups, heart rate differed significantly, with the analgesic group having a nearly 10 bpm higher heart rate (*p* = 0.004). While serum amylase levels did not differ significantly, the analgesic group had serum lipase levels over 120 U/L higher than the nonanalgesic group (*p* = 0.018). Additionally, WBC and hemoglobin levels were significantly higher in the analgesic group. The median hospital stay was 8 days in the analgesic group compared to 6 days in the nonanalgesic group, with a statistically significant difference (*p* < 0.001).

**TABLE 1:** Baseline characteristics of the 2 groups.

Variables	Group		*p* value
	Nonanalgesic (*n* = 82)	Analgesic (*n* = 69)	
Age (y)^a^	44 (36–54)	41 (32–49)	0.096^b^
Gender, *n* (%)			0.352^c^
Male	57 (69.5)	43 (62.3)	
Female	25 (30.5)	26 (37.7)	
BMI, (mean ± SD)	25.1 ± 3.6	25.3 ± 3.9	0.694^d^
Maximum pain score at admission, *n* (%)			0.029^e^
0	10 (12.2)	3 (4.3)	
1–3	64 (78.0)	50 (72.5)	
4–7	8 (9.8)	16 (23.2)	
8–10	0 (0)	0 (0)	
Time of presentation, *n* (%)			0.262^c^
00:00–7:59	7 (8.5)	12 (17.4)	
08:00–15:59	39 (47.6)	30 (43.5)	
16:00–23:59	36 (43.9)	27 (39.1)	
First episode,*n* (%)			0.32^c^
Yes	54 (65.9)	40 (58.0)	
No	28 (34.1)	29 (42.0)	
Etiology of AP, *n* (%)			0.041^c^
Gallstone related	15 (18.3)	13 (18.8)	
Alcohol related	8 (9.8)	8 (11.6)	
Triglyceride related	16 (19.5)	26 (37.7)	
Others	43 (52.4)	22 (31.9)	
Heart rate (bpm)^a^	81.5 (72–98)	91 (80–108)	0.004^b^
SBP, mm Hg, (mean ± SD)	133.4 ± 18.9	136.7 ± 18.6	0.415^d^
DBP, mm Hg, (mean ± SD)	87.0 ± 12.9	86.8 ± 12.3	0.921^d^
Severity of pancreatitis, *n* (%)			< 0.001^c^
Mild	71 (86.6)	40 (58.0)	
Moderately severe	6 (7.3)	14 (20.3)	
Severe	5 (6.1)	15 (21.7)	
Length of hospital stay (days)^a^	6 (5–8)	8 (6–12)	< 0.001^b^
Hemoglobin (g/dL) (mean ± SD)	142.4 ± 25.0	150.5 ± 22.2	0.038^d^
WBC count (/mm^3^)^a^	10.2 (8.0–13.3)	12.3 (10.0–15.1)	0.01^b^
Total bilirubin (μmol/L)^a^	15.8 (10.6–25.6)	13.7 (10.8–20.3)	0.271^b^
ALT (U/L)^a^	28.5 (13.2–58.7)	31.7 (19.0–61.3)	0.348^b^
AST (U/L)^a^	23.9 (16.3–43.3)	27.0 (21.4–48.0)	0.195^b^
Serum amylase (U/L)^a^	138.5 (55.4–387.4)	174.6 (78.3–484.5)	0.095^b^
Serum lipase (U/L)^a^	257.7 (70.8–622.4)	371.6 (130.9–1317.3)	0.018^b^
C‐reactive protein (mg/L)^a^	35.3 (10.3–106.3)	52.8 (16.9–138.2)	0.198^b^
Level of education, *n* (%)			0.677^e^
Illiterate	1 (1.2)	0 (0)	
Elementary Education	8 (9.8)	9 (13.0)	
Secondary Education	51 (62.2)	38 (55.1)	
Higher Education	22 (26.8)	22 (31.9)	

*Note:* ALT, alanine aminotransferase; AST, aspartate aminotransferase.

Abbreviations: AP, acute pancreatitis; BMI, body mass index; DBP, diastolic blood pressure; SBP, systolic blood pressure; SD, standard deviation; WBC, white blood cell.

^a^Values expressed as median and interquartile range.

^b^Wilcoxon rank‐sum test.

^c^Chi‐square tests.

^d^t‐test.

^e^Fisher’s exact test.

Except for length of hospital stay, the seven variables with statistically significant differences in univariate analysis were further included in multivariate logistic regression analysis. Table 2 shows that compared to a pain score of 0 at admission, a score of 4–7 was associated with a 5.86‐fold increased risk of analgesic use (odds ratio [OR] = 6.86, 95% confidence interval [CI]: 1.12–41.99, *p* = 0.037). Moderately severe and severe AP (vs. mild) were associated with 2.94‐fold (OR = 3.94, 95% CI: 1.19–12.98, *p* = 0.024) and 4.05‐fold (OR = 5.05, 95% CI: 1.22–20.8, *p* = 0.025) increased risk of analgesic use, respectively. In binary logistic regression analysis, we divided the serum lipase level of all patients into four levels (< 108.1 U/L; ≥ 108.1 U/L‐< 293.4 U/L; ≥ 293.4 U/L ‐< 809.6 U/L; ≥ 809.6 U/L) based on the IQR. Although 108.1 U/L ≤ serum lipase level < 293.4 U/L and 293.4 U/L ≤ serum lipase level < 809.6 U/L (vs. < 108.1 U/L) did not significantly increase analgesic use risk, patients with serum lipase level ≥ 809.6 U/L had a 2.8‐fold increased risk (OR = 3.8, 95% CI: 1.27–11.37,*p* = 0.017).

**TABLE 2:** Results of binary logistic regression analysis for factors associated with analgesic use.

Variable	*B* (SE)	*p* value	OR [95% CI]
*Maximum pain score at admission*			
0	—	—	1
1–3	1.249 (0.771)	0.105	3.49 [0.77–15.82]
4–7	1.926 (0.924)	0.037	6.86 [1.12–41.99]

*Etiology of AP*			
Gallstone related	—	—	1
Alcohol related	0.015 (0.728)	0.984	1.02 [0.24–4.22]
Triglyceride related	0.412 (0.612)	0.501	1.51 [0.46–5.00]
Others	−0.080 (0.551)	0.885	0.92 [0.31–2.72]

*Severity of pancreatitis*			
Mild	—	—	1
Moderately severe	1.370 (0.609)	0.024	3.94 [1.19–12.98]
Severe	1.619 (0.723)	0.025	5.05 [1.22–20.80]

Heart rate	0.007 (0.011)	0.53	1.01 [0.99–1.03]
Hemoglobin	0.002 (0.009)	0.871	1.00 [0.98–1.02]
WBC count	−0.005 (0.050)	0.916	1.00 [0.90–1.10]

*Serum lipase level (U/L)*			
< 108.1	—	—	1
≥ 108.1–< 293.4	0.962 (0.544)	0.077	2.62 [0.90–7.61]
≥ 293.4–< 809.6	0.380 (0.541)	0.482	1.46 [0.51–4.23]
≥ 809.6	1.335 (0.559)	0.017	3.80 [1.27–11.37]

Abbreviations: AP, acute pancreatitis; CI, confidence interval; OR, odds ratio; SE, standard error; WBC, white blood cell.

Disease severity, WBC, and frequency of analgesic use were significantly associated with prolonged hospital stay, particularly disease severity and analgesic use (Table 3).

**TABLE 3:** Results of multiple linear regression analysis for factors associated with increased length of stay.

Variables	Unstandardized coefficients		Standardized coefficients	*p* value
	Beta	Std. error	Beta	
Severity of AP	2.510	0.684	0.298	< 0.001
Etiology of AP	−0.378	0.388	−0.071	0.332
Hemoglobin	−0.038	0.020	−0.151	0.058
WBC count	0.237	0.109	0.177	0.032
Serum lipase	0.697	0.390	0.131	0.076
Maximum pain score at admission	−1.819	0.945	−0.149	0.056
Frequency of analgesics used	0.407	0.133	0.247	0.003

Abbreviations: AP, acute pancreatitis; WBC, white blood cell.

## 4. Discussion

This study revealed that, in addition to the previously identified associations of baseline pain level and AP severity with analgesic use, there is also a significant association between high serum lipase level and analgesic demand in hospitalized patients with AP.

Previous research on factors associated with analgesic use in AP is limited. It is suggested that radiological severity of AP correlates positively with opioid dose requirements [18]. Parsa N et al. found that early hemoconcentration (hematocrit ≥ 44%) is associated with increased opioid demand in hospitalized AP patients [19]. Other studies indicate that admission pain severity, systemic inflammation, etiology of pancreatitis, sex, race, and institution of treatment are associated with opioid use [14].

Our results show that patients with moderate‐to‐severe pain (score 4–7) at admission had a 5.86‐fold higher risk of analgesic use compared to those with no pain. However, mild pain (score 1–3) did not significantly increase analgesic use risk, suggesting limitations in the clinical utility of pain assessment tools. Clinicians should be cautious about discrepancies between patients’ pain reports and actual analgesic needs, particularly at low pain scores. Pain assessment should adopt a multidimensional approach, incorporating objective indicators (e.g., serum lipase and imaging) alongside subjective scores.

Patients with SAP often experience more severe abdominal pain and require higher opioid doses [20]. Our findings support this, as moderately severe and severe AP patients were more likely to receive analgesics. However, analgesics may exacerbate AP. A prior animal study on AP models demonstrated that fentanyl pretreatment worsened pancreatic necrosis, while ibuprofen pretreatment increased pancreatic edema [21]. Clinical studies also suggest some opioid drugs may increase the risk of local complications or severe adverse events [22, 23]. Thus, the relationship between AP severity and analgesic use warrants further investigation.

Some studies have focused on associations between serum lipase level and AP prognosis or etiology. Coffey et al. found that elevated lipase within 24 h of presentation may be a simple clinical predictor of severe AP in children [24], and other evidence also suggests a potential correlation between serum lipase and AP severity, particularly with very high thresholds or in specific patient subgroups [25–27]. In contrast, other multiple studies concluded that lipase levels alone had limited utility in assessing prognosis or severity [28–31]. Therefore, it remains difficult to draw a definitive conclusion as to whether the observed association between elevated serum lipase levels and increased analgesic use is simply a consequence of higher lipase reflecting pancreatic necrosis and greater disease severity. Nevertheless, because this possibility exists, our findings should still be interpreted with caution. Few studies have explored the association between lipase elevation and analgesic use. Our finding that a lipase level ≥ 809.6 U/L is associated with analgesic use provides a clue for AP pain management. Clinicians may prioritize early pain assessment and individualized regimens (e.g., potent opioids or NSAIDs combinations) in these patients to avoid under‐ or overtreatment.

Although significant differences in heart rate, WBC and etiology distribution were observed between the two groups, these did not independently predict analgesic use. This contrasts with another study [14], highlighting the complexity and variability of AP pain management. Future research should further explore these indicators’ relationships with analgesic demand.

Our results also indicate that disease severity, WBC count, and analgesic use were associated with prolonged hospitalization, likely reflecting the impact of disease complexity on treatment duration. Importantly, this underscores the need to address analgesic demands to reduce hospital stays.

In the current era of opioid misuse prevention and growing evidence supporting comparable efficacy between opioids and NSAIDs for AP pain [12, 15, 32, 33], tailoring analgesic regimens to patient needs has become critical. While guidelines exist for cancer and postoperative pain, AP pain management involves multiple specialties (emergency, internal medicine, and surgery), complicating standardization and contributing to heterogeneity in analgesic use, particularly opioids. Significant racial and global disparities in AP analgesic practices have been reported [13, 34]. Both insufficient and excessive use of analgesic medications are clinically inappropriate for patient care. Some studies advocate for individualized and effective AP analgesic strategies to prevent chronic pain syndromes, favoring step‐down (vs. step‐up) approaches [10]. Our study provides clues for individualized AP pain management. Future research could integrate lipase level, imaging features, or other clinical markers to develop predictive scoring systems, ensuring safe and appropriate analgesic use in AP patients. It must be stressed that while predictive markers such as lipase levels may help anticipate analgesic needs, they do not replace careful dynamic pain assessment and management, and there is no high‐quality evidence for withholding opioids from patients—including children—experiencing severe pain. We consistently uphold that analgesic therapy—including opioids when clinically indicated—should be tailored to individual patient needs and pain severity.

This study has several limitations. First, the small sample size led to wide OR confidence intervals (e.g., 1.12–41.99 for the pain score 4–7 in the logistic regression analysis). Second, this study did not analyze the dosage of analgesics, as the total analgesic dose would more accurately reflect patients’ pain management needs. However, in clinical practice, the wide variety of analgesics used—including different NSAIDs and other types of nonopioid pain medications—makes standardized dose calculation impractical for some drug categories. Third, the study did not include patients with severe pain (pain score 8–10) upon admission, which may lead to a biased or incomplete conclusion. The likely reason is that the majority of AP patients were admitted through the emergency department, where analgesic treatment had often already been administered to these patients with severe pain before transferring them to inpatient units. Moreover, while our logistic regression analysis statistically controlled for several important clinical confounders—including disease severity, white blood cell count, and hemoglobin levels—and confirmed the independent predictive value of elevated lipase, there are other potential confounders that were not included. This study did not precisely record the exact timing of analgesic administration relative to serum lipase measurement (e.g., whether analgesics were given immediately before or after the blood draw). This ambiguity in the temporal relationship makes it challenging to strictly infer the association between elevated lipase and analgesic need. Future prospective studies should standardize the timing of analgesic intervention relative to the serum lipase measurement window. Finally, the single‐center retrospective design may constrain the extrapolation of the conclusions. It is crucial to emphasize that analgesic use could also be influenced by variability in clinicians’ preferences—including subjective differences in pain assessment and prescribing habits—and that this variability represents a significant yet difficult‐to‐quantify confounder. This complexity thereby highlights the big challenges inherent in designing and conducting clinical trials targeting therapeutic strategies for AP‐related pain [35].

In conclusion, high serum lipase level (≥ 809.6 U/L) is associated with increased analgesic use in hospitalized patients with AP, providing potential clinical implications for optimizing analgesic strategies in AP care. However, the potential influences of the discussed confounders, including disease severity, timing of analgesic usage, and clinician preference variability, necessitate cautious interpretation of the results, and further large‐scale clinical studies are needed to confirm its clinical importance and applicability, particularly the predictive efficacy of lipase level combined with other clinical indicators.

## Author Contributions

Liangqing Gao: conceptualization, data collection, and writing; Ruifang Zhang: reviewing, editing, and data collection; Yizhi Xiao: data collection and reviewing; Chengmin Ma: data collection, analysis and interpretation of data; Xiaofeng Li: supervision and reviewing.

## Funding

No funding was received for this work.

## Disclosure

All authors have agreed to the final submitted version.

## Ethics Statement

The study protocol was reviewed and approved by the Ethics Review Committee of our hospital (reference number: 2025‐061).

## Consent

Due to the retrospective and anonymous nature of the data, the informed consents were waived.

## Conflicts of Interest

The authors declare no conflicts of interest.

## Data Availability

The data that support the findings of this study are available on request from the corresponding author.
